# Electrostatic interactions between single arginine and phospholipids modulate physiological properties of sarcoplasmic reticulum Ca^2+^-ATPase

**DOI:** 10.1038/s41598-022-16091-9

**Published:** 2022-07-16

**Authors:** Kazuo Yamasaki, Takashi Daiho, Satoshi Yasuda, Stefania Danko, Jun-ichi Kawabe, Hiroshi Suzuki

**Affiliations:** Department of Biochemistry, Asahikawa Medical University, Midorigaoka-higashi 2-1-1-1, Asahikawa, 078-8510 Japan

**Keywords:** Membrane proteins, Enzyme mechanisms, Ion transport, Membrane lipids, Bioenergetics

## Abstract

Arg324 of sarcoplasmic reticulum Ca^2+^-ATPase forms electrostatic interactions with the phosphate moiety of phospholipids in most reaction states, and a hydrogen bond with Tyr122 in other states. Using site-directed mutagenesis, we explored the functional roles of Arg324 interactions, especially those with lipids, which at first glance might seem too weak to modulate the function of such a large membrane protein. The hydrogen bond forms transiently and facilitates Ca^2+^ binding from the cytoplasmic side. The contributions of the electrostatic interactions to the reaction steps were quantified using a rate *vs* activity coefficient plot. We found that the interaction between Arg324 and lipids decreases the affinity for luminal Ca^2+^. The transformation rate of the phosphoenzyme intermediate is facilitated by the electrostatic interactions, and the function of these interactions depends not only on the type but also on the composition of the phospholipids. The properties observed in microsomes could not be reproduced with any single phospholipid, but with a mixture of phospholipids that mimics the native membrane. These results suggest the importance of swapping of the lipid partners of different headgroups in the reaction step. This study shows that Arg324 plays a role in the reaction cycle via complex intra-protein and protein-lipid interactions.

## Introduction

Membrane proteins are anchored to the membrane in various ways; for example, via hydrophobic α-helices penetrating the membrane, hydrophobic protein surfaces, long lipid chains covalently bound to protein residues, or electrostatic anchoring between positively charged basic amino acid residues of the protein (lysine or arginine) and negatively charged anionic lipid headgroups^1–3^. In some cases, the formation and disruption of such interactions are under precise control and elicit dynamic changes to population distribution of conformers. These changes are often associated with on/off enzyme states or signal transduction and are easily investigated^4^. However, it is more difficult to demonstrate the role of noncovalent interactions between lipids and single amino acid residues in large membrane proteins, where a particular interaction with the membrane may seem trivial in the context of the whole protein, and the precise structure at the interface is not available. Tightly bound lipid molecules have been found at the surface or in grooves between transmembrane α-helices in some membrane proteins^5^; however, examples of direct interactions between particular protein residues and lipid headgroups are rare, and demonstrations of their functional significance still rarer^6,7^. Therefore, further exploration of the effect of microscopic protein-lipid interactions on the total properties of large membrane proteins is necessary for a comprehensive understanding of membrane protein's function.

**Figure 1:**
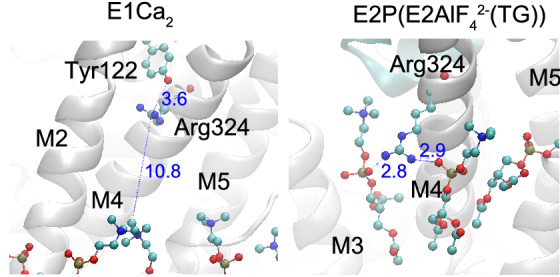
Protein-phospholipid interaction observed in crystal structures. Residue Arg324 and neighboring phospholipids in E1Ca_2_ (PDB ID: 5XA7) and an E2P analog (E2AlF_4_^2−^(TG); PDB ID: 5XA9) are shown in ball and stick models. Residue Tyr122 is also shown in the E1Ca_2_ structure. The distance between Arg324 and the phosphate moiety is larger than 10 Å in E1Ca_2_. Protein-phospholipid interactions between Arg324 and the phosphate groups of phosphatidylcholines observed in the E2P analog are indicated by blue dotted lines. The distances (Å) are indicated by blue numbers.

Sarcoplasmic reticulum Ca^2+^-ATPase (SERCA1a) is a member of the P-type ion transport ATPase family, which forms a phosphoenzyme intermediate (EP) during the reaction cycle (Supplemental Fig. S1). Ca^2+^ binding sites of SERCA1a face the cytoplasmic side with high affinity in the E1Ca_2_ state, and the luminal side with low affinity in the E2PCa_2_ state. These asymmetrical affinities for Ca^2+^ allow the pump to transport Ca^2+^ from the cytoplasm to the lumen against a concentration gradient of more than 1000-fold. This large membrane protein (approx. 110 kDa) possesses ten membrane helices (M1–10) and three cytoplasmic domains (A, P, and N). The structures of most reaction intermediates have been elucidated and the motion of cytoplasmic domains resulting in the rearrangement of transmembrane helices is now understood^8–12^. In 2017, Toyoshima’s group identified the locations of all phospholipids surrounding SERCA1a in four enzymatic states^13^ and showed that several amino acid residues form electrostatic interactions with charged headgroups of phospholipids. Arg324 was shown to interact with the phosphate moiety of phosphatidylcholine (PC) in three of the four enzyme states (Fig. 1, Supplementary Fig. S2 and S3). This residue is distant from the membrane in the E1Ca_2_ state (> 10 Å) and close to the OH moiety of Tyr122 (< 3 Å) on top of the long M2 helix. The formation of E1PCa_2_ results in Arg324 descending toward the membrane, with inclination of the M4 helix, and establishing an electrostatic interaction with a phosphate group of a phospholipid. The interaction is rearranged by the conformational change that accompanies the transition from E1PCa_2_ to E2PCa_2_ or E2P (steps 4 and 5 in Supplementary Fig. S1; see also Fig. 1, Supplementary Fig. S2 and S3). This is the rate-limiting step of the overall reaction cycle of the Ca^2+^-ATPase. The interaction seems to strengthen as judged by an increase in the number of participating lipid molecules. The existence of an interaction between Arg324 and lipids in the E1PCa_2_ and E2P states has also been shown by molecular dynamics simulation of SERCA1a in a dioleoyl phosphatidylcholine bilayer^14^. In the following step (i.e., E2P to E2) (step 6 in Supplementary Fig. S1), the interaction is maintained but seems to weaken by the loss of one engaged phospholipid. Finally, the interaction is completely disrupted upon Ca^2+^ binding to the Ca^2+^-ATPase (E2 to E1Ca_2_), and Arg324 ascends to interact with Tyr122 (Fig. 1 and Supplementary Fig. S3).

Substitution of Arg324 by Ala or Glu changes the enzymatic properties of Ca^2+^-ATPase^15^. The EP transitions (E1PCa_2_ → E2PCa_2_) of those mutants are slower than those of the wild-type protein, and the reactivation steps (E2 → E1Ca_2_) twice as fast. These observations suggest that the interaction, which is rearranged in the phosphoenzyme transition step (E1PCa_2_ to E2P) and broken in the reactivation step (E2 to E1Ca_2_), has a functional significance. However, the lipid molecules surrounding SERCA possess high fluidity^16^ and the presence of a couple of protein-lipid electrostatic interactions seems negligible comparing with the large conformational changes of SERCA. Therefore, it should be verified whether these interactions modulate the function of SERCA1a and the physiological role of SERCA.

To clarify the possible role of the interactions between Arg324 and phospholipids, we explored the use of nanodiscs^17–19^ formed with Ca^2+^-ATPase and different lipids^20^. A plot of the logarithm of the EP transition rate versus the square of the mean activity coefficient^20,21^ quantifies the contribution of electrostatic interactions between Arg324 and lipid headgroups in the EP transition step. Our study shows that Arg324-lipid interactions decrease the apparent affinity for luminal Ca^2+^. Additionally, it reveals that Arg324 facilitates the Ca^2+^ binding step (from E1 to E1Ca_2_) by establishing a hydrogen bond with Tyr122. Furthermore, the complex rearrangement process of different phospholipids in Arg324-lipid interactions influences the EP transition step.

## Results

### SERCA1a ATPase activity and affinity for cytoplasmic or luminal Ca^2+^

**Figure 2:**
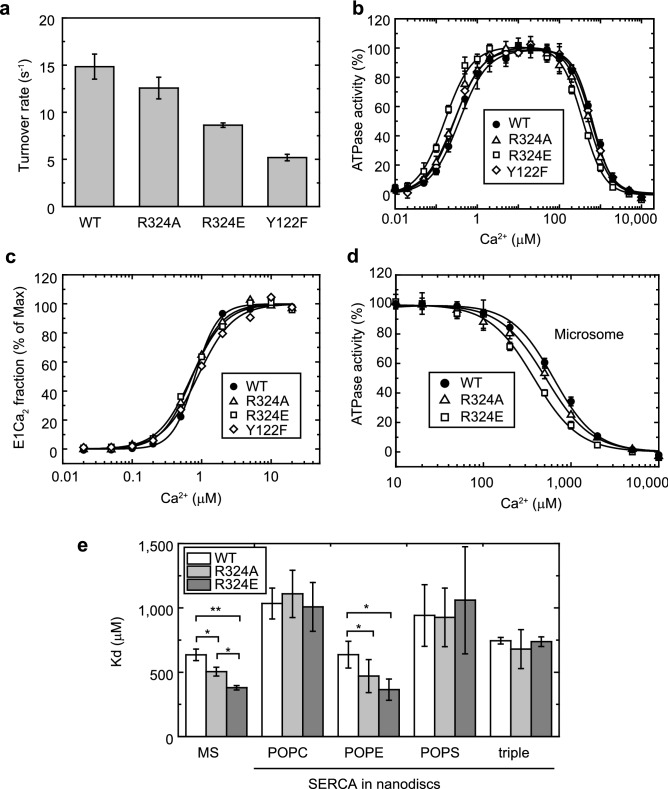
Ca^2+^ dependence of ATPase activity and affinity for cytoplasmic and luminal Ca^2+^. (**a**) Turnover rates of Ca^2+^ dependent ATP hydrolysis of microsomes prepared from COS-1 cells expressing SERCA1a proteins. The ATP hydrolysis rate measured in the absence of Ca^2+^ (2 mM EGTA) was subtracted from the rate measured in the presence of 10 μM Ca^2+^; the obtained Ca^2+^ dependent ATP hydrolysis rates were divided by the amount of phosphorylation sites. (**b**) Ca^2+^ dependence of ATP hydrolysis rates of WT (●), R324A (△), R324E (☐), and Y122F (◇) were measured. The activity was normalized to the maximum level of ATPase activity as 100% and ATPase activity in the absence of Ca^2+^ as 0%. The solid lines show the least square fit to a complex of two Hill equations (see legend for Supplementary Table S1): increase in lower Ca^2+^ range and decrease in higher Ca^2+^ range. The parameters are listed in Supplementary Table S1. (**c**) Ca^2+^-binding curves of expressed SERCA1a estimated from EP formation activity. The dissociation constants obtained from the fitting curves are listed in Supplementary Table S2. (**d**) Enlarged view of the ATPase activity plot shown in **b** in the range of 10 to 10,000 μM Ca^2+^. (**e**) Estimated dissociation constants for luminal Ca^2+^ of SERCA1a in microsomes or nanodiscs. Labels in abscissa indicate the conditions of SERCA1a. MS, microsomes prepared from COS-1cells; POPC, palmitoyl-oleoyl-phosphatidylcholine; POPE, palmitoyl-oleoyl-phosphatidylethanolamine; POPS, palmitoyl-oleoyl-phosphatidylserine; “triple,” mixture of POPC, POPE, and POPS. Values indicate the mean ± standard deviation from at least three experiments. *P < 0.05. **P < 0.005. The Kd and Hill coefficient values are listed in Supplementary Table S5.

Microsomes prepared from COS-1 cells transfected with SERCA1a cDNA were used for kinetic assays. The experiments were performed in the presence of calcium ionophore A23187; therefore, the microsomes were no longer sealed for Ca^2+^. The turnover rates of Ca^2+^-dependent ATP hydrolysis of the wild-type and mutant proteins were determined in the presence of 10 μM Ca^2+^ (Fig. 2a). The rates of mutants are approximately 85%, 58%, and 35% that of the wild-type for R324A, R324E, and Y122F, respectively. The Ca^2+^ concentration dependences of ATPase activities show bell-shaped profiles (Fig. 2b), indicating that both Ca^2+^-binding sites facing the cytoplasmic and luminal sides are accessible under these conditions. The half effective concentrations (K_0.5_) of Ca^2+^ for activation and inhibition of the ATPase activity in the wild-type are 0.36 μM and 0.64 mM, respectively (Supplementary Table S1). The K_0.5_ values of activation are slightly lower than that of the wild-type. Additionally, our results confirm our previous report showing that affinities for cytoplasmic Ca^2+^ estimated from Ca^2+^ dependency of EP-formation activity were unchanged in these mutants^15^ (Fig. 2c, Supplementary Table S2). The slower EP transition rates of the mutants^15^ would cause shifts of the K_0.5_ of activation ATPase activities towards a lower range. Affinities for luminal Ca^2+^ (K_0.5_ of the inhibition by luminal Ca^2+^) were also altered by substitutions of Arg324 (Fig. 2b,d and Supplementary Table S1). Both R324A and R324E possess higher affinity for luminal Ca^2+^ than the wild-type protein; that of Y122F is nearly identical to that of the wild-type.

To explore the effects of lipid headgroups on the affinity for luminal Ca^2+^ of Ca^2+^-ATPase, wild-type and Arg324 mutants from microsomes were reconstituted in nanodiscs harboring palmitoyl-oleoyl-phosphatidylcholine (POPC), palmitoyl-oleoyl-phosphatidylethanolamine (POPE), palmitoyl-oleoyl-phosphatidylserine (POPS), or a mixture of these three lipids^20^ (Fig. 2e). SERCA1a reconstituted in POPC or POPS had lower affinity for luminal Ca^2+^ and the values were not altered by the Arg324 mutations. In contrast, SERCA1a reconstituted in POPE had the same affinity for luminal Ca^2+^ as SERCA1a in microsomes. This suggests that Arg324 interacts with a phosphatidylethanolamine (PE) headgroup under native conditions and not with PC as observed in a crystal structure^13^. When SERCA1a was reconstituted in a mixture of the three phospholipids, which mimics the lipid component of the natural sarcoplasmic reticulum (SR) membrane^22^, the affinity for luminal Ca^2+^ was similar to that of the wild-type in microsomes. However, unlike the pump in microsomes, the affinity was not altered by the Arg324 mutations (“triple” in Fig. 2e).

### Role of Arg324-Tyr122 or Arg324-lipid interactions in the Ca^2+^ binding step from the cytoplasmic side

**Figure 3:**
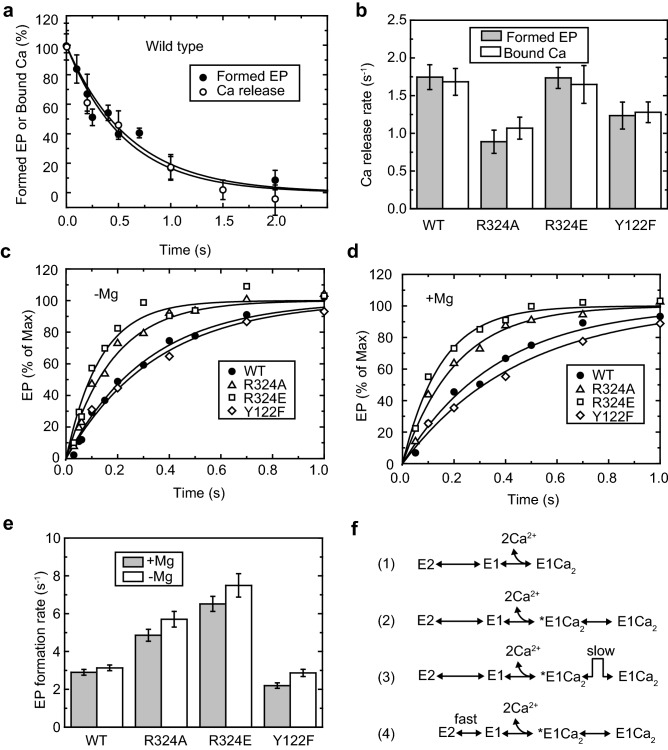
Kinetic analyses of the dissociation and association processes of cytoplasmic Ca^2+^. (**a**) Time courses of Ca^2+^ release from E1Ca_2_ in microsomes prepared from COS-1 cells expressing wild-type SERCA1a, estimated from EP formation activity (closed symbols) or bound Ca^2+^ (open symbols) at 4 °C. The solid lines shows the least squares fit to a single exponential decay. (**b**) Rate constants of Ca^2+^ release from SERCA1a wild-type or mutants obtained from **a** (± standard deviation) from at least three experiments. The gray bars indicate estimated values from formed EP and the open bars from bound Ca^2**+**^. (**c, d**) Microsomes (20 μg/ml) were incubated in medium containing 50 mM MOPS/Tris (pH 7.0) and 0.1 M KCl in the presence of 7 mM MgCl_2_ and 1.2 mM EGTA (**c**) or 0.2 mM EDTA without MgCl_2_ (**d**) at 4 °C. EP formation was initiated by addition of medium containing MgCl_2_, CaCl_2_, and [γ-^32^P]ATP. Final concentrations of Mg^2+^, Ca^2+^, and ATP in the reaction medium were 7 mM, 0.4 mM, and 10 μM, respectively. The reaction was terminated with 7% TCA (final concentration) at the indicated time points after ATP addition and the amount of EP was measured. The solid lines show the least squares fit to a single exponential increase from 0 time. (**e**) Rate constants obtained from **c** and **d** (± standard error of the mean). The parameters are listed in Supplementary Table S3. (**f**) Reaction schemes of the E2 to E1Ca_2_ transition step.

The time course of Ca^2+^ release from wild-type and mutant E1Ca_2_ was measured using two different methods (Fig. 3a,b). In the first method, using the direct determination of bound ^45^Ca^2+^ (open circles and bars in Fig. 3a,b, respectively), Ca^2+^-ATPase was incubated with ^45^Ca and spotted on a membrane filter. Then, Ca^2+^ release was initiated by washing the filter with a buffer containing EGTA. At various time points, the membrane was washed with a buffer containing 10 mM cold CaCl_2_ and 0.1 mM ATP. As previously shown, this buffer changes the Ca^2+^-ATPase with remaining bound radioactive Ca^2+^ into E1PCa_2_ and washes out unbound ^45^Ca^2+^^23^. In the second method, using the indirect determination from EP (closed circles in Fig. 3a and gray bars in Fig. 3b), Ca^2+^-ATPase was incubated with non-radioactive Ca^2+^, and Ca^2+^ release was initiated by EGTA treatment. At various time points, the remaining E1Ca_2_ was phosphorylated by adding AT^32^P and the amount of EP thus formed was measured. Using this method, the release of the first Ca^2+^ can be observed because E1Ca (i.e., E1 bound to only one Ca^2+^) is no longer able to form EP. The Ca^2+^ release time courses measured using these two methods were in complete agreement (Fig. 3a,b), indicating that the release rate of the second Ca^2+^ is too fast to distinguish from the first Ca^2+^ release or that there is a transition step before Ca^2+^ release from E1Ca_2_, which is much slower than Ca^2+^ release. The Ca^2+^ release rate of the R324E mutant, where a hydrogen bond can form between the substituted Glu and Tyr122, is the same as that of the wild-type. On the other hand, the release rates of R324A and Y122F, where a hydrogen bond cannot be established, were slower than that of the wild-type (Fig. 3b). These results indicate that the hydrogen bond between Arg324 and the hydroxyl group of Tyr122 facilitates Ca^2+^ release from E1Ca_2_. However, the mutations did not affect the affinity for cytosolic Ca^2+^ (Fig. 2c and Supplementary Table S2), indicating that the bond in this step is transient.

The time course of EP formation starting from Ca^2+^-unbound species (E1 or E2) (Fig. 3c,d) explores the rate of the E2-E1Ca_2_ transition (steps 1 and 2 in Supplementary Fig. S1), which may include Mg^2+^ binding and dissociation steps^24,25^. In fact, an E1Mg structure is known from crystal analysis^26,27^. Ca^2+^-ATPase was preincubated in a Ca^2+^-free medium in the presence or absence of Mg^2+^, and EP formation was initiated by addition of Mg^2+^, ATP, and Ca^2+^. The EP formation rate under these conditions was much slower than that of Ca^2+^-preincubated Ca^2+^-ATPase (from E1Ca_2_, > 20 s^−1^). The Ca^2+^ concentration (400 μM) was more than 500-times higher than the Ca^2+^ dissociation constants (approximately 0.8 μM; Fig. 2c and Supplementary Table S2). Therefore, the rate of Ca^2+^ association to E1 should be much faster than the rate of Ca^2+^ dissociation from E1Ca_2_ (1–2 s^−1^; Fig. 3b). The EP formation rates of the wild-type and mutants were not considerably affected by Mg in the preincubation medium. Therefore, the EP formation rates observed in this measurement reflect the step before E1 formation (E2 to E1, step 1 in Supplementary Fig. S1). This step was accelerated in both R324A and R324E but not in Y122F (Fig. 3c,d).

### Contribution of Arg324-phospholipid electrostatic interaction on EP transition step

**Figure 4:**
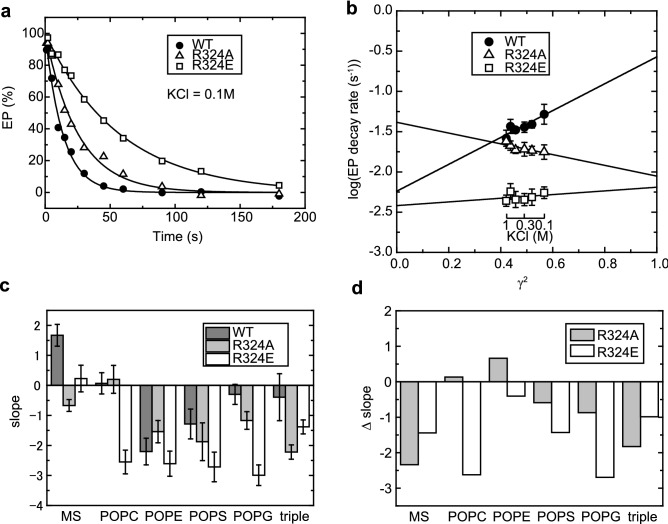
Relationship between logarithm of EP transition rates of expressed Ca^2+^-ATPases and square of mean activity coefficient *γ*_±_^2^. (**a**) EP transition time courses of microsomes were measured in the presence of 0.1 M KCl at 0 °C. Microsomes were incubated in 10 μM CaCl_2_. Then the mixture was mixed with the medium containing [γ-^32^P]ATP and EGTA. Finally, the reaction mixture contained 50 mM MOPS/Tris (pH 7.0), 0.1 M KCl, 7 mM MgCl_2_, 2 mM EGTA, 5 μM CaCl_2_ and 10 μM [γ-^32^P]ATP (free Ca^2+^  < 1 nM). The reaction was terminated by 7% TCA at the indicated time after ATP-EGTA addition and the amount of EP was measured. The solid lines show the least squares fit to a single exponential decay. (**b**) EP transition rates were determined with wild type and mutants in various concentrations of KCl as indicated, and their logarithms are plotted versus *γ*_±_^2^ as previously described^21^. The values presented are the mean ± SD (n = 3–5). Solid lines show the least square fit in a linear regression, and the fitting parameters (± SEM), the slope and the intercept at *γ*_±_^2^ = 0, are listed in Supplementary Table S4. (**c**) from rate versus activity coefficient plot of Ca^2+^-ATPase embedded in nanodiscs harboring various phospholipids are shown as bar graphs (see Supplementary Fig. S4). The error bars indicate ± SEM. The parameters are also listed in Supplementary Table S4. (**d**) The difference between the slopes of wild type and mutants (Δslope) are shown.

The EP transition step (from E1PCa_2_ to E2PCa_2_) is the rate-limiting step of the overall reaction cycle of the Ca^2+^-ATPase. Therefore, the decay rate of EP formed from ATP can be considered as the rate of the EP transition. The EP transitions of the mutants in the presence of 0.1 M KCl were slower than that of the wild-type (Fig. 4a). The extents of suppression in the rates were larger than those observed for Ca^2+^-ATPase activity (Fig. 2a). This discrepancy may arise from the difference in temperature (0 °C for EP transition and 25 °C for Ca^2+^-ATPase activity).

We used a plot of the logarithm of the EP transition rate versus the square of the mean activity coefficient^21^ to quantify the contribution of electrostatic interactions. The logarithm of the rate is a linear function of activation energy. The electrostatic energy between interacting charges is expected to be proportional to the product of the activities of the charges and, therefore, to the square of the mean activity coefficient γ_±_^2^. A plot of the logarithm of the rate versus γ_±_^2^ gives a linear relationship and thereby divides the activation energy into two components: an electrostatic force and a non-electrostatic force. The slope reflects the amplitude of the contribution of the electrostatic energy, and the intercept at γ_±_^2^ = 0 reveals the contribution of the non-electrostatic energy because these are conditions in which the electrostatic interactions that can be screened on the protein surface are completely shielded by salt. As the mean activity coefficient (γ_±_) can be altered by changing the ionic strength of the solution, we applied this approach to the EP transition rates measured in 0.1–1 M KCl (Fig. 4b).

Wild-type Ca^2+^-ATPase showed a line with a positive slope. This indicates that electrostatic interactions significantly contribute to the EP transition step and have an overall accelerating effect, as previously reported^21^. The Ala and Glu substitutions of Arg324 have a profound lowering effect on the slope, with that of the Ala substitution being negative (Fig. 4b, open symbols). This indicates that both substitutions diminish the normally favorable electrostatic interactions or introduce new unfavorable electrostatic interactions into the EP transition step. The greater substitution effect of Ala over Glu may indicate that the change in the charged side-chain (Arg to Glu) results in partial compensation through other electrostatic interactions. These mutations also altered intercept values. The Ala substitution increased the intercept value, whereas the Glu substitution slightly decreased the value. These results imply that the smaller Ala relieves unfavorable steric constraints, unlike the larger Glu.

Wild-type and mutants (R324A and R324E) were reconstituted in nanodiscs harboring POPC, POPE, POPS, palmitoyl-oleoyl-phosphatidylglycerol (POPG), or a mixture of POPC, POPE and POPS. EP transition rates were measured under various KCl concentrations (Supplementary Fig. S4). The fitting parameters of the rates *vs* activity coefficient plots are listed in Supplementary Table S4. The obtained slope values are also shown in a bar graph (Fig. 4c) and the difference in the slope values between mutants and wild-type are shown in Fig. 4d. The intercept values are shown in Supplementary Fig. S5.

If the electrostatic interaction between Arg324 and the phosphate group of phospholipids has positive effects on the EP transition, the order of slopes values would be WT > R324A > R324E. However, as mentioned above, the order of the slope value in microsomes is WT > R324E > R324A (Fig. 4c,d and Table 1). When SERCA1a was embedded in neutral phospholipids (POPC or POPE), the slope value for R324A was larger than that of R324E, as expected. However, the slope value for the wild-type was slightly (POPC) or substantially (POPE) smaller than that of R324A. On the other hand, the expected order was observed in the samples possessing negatively charged acidic phospholipids (POPS or POPG). The order of the slope values of SERCA1a in any nanodiscs with a single type of phospholipid differed from that of microsomes, the value for R324A being larger than that of R324E. However, SERCA1a embedded in a mixture of the three types of lipids in a proportion similar to that of SR vesicles showed the same order of slope values as microsomes (Fig. 4c,d and Table1). Similar trends were also observed in intercept values (Supplementary Table S4 and Supplementary Fig. S5). In microsomes, the intercept value of R324A was larger than that of the wild-type; that of R324E was slightly smaller. This order was also observed in SERCA1a embedded in the mixture of three types of lipids but not in SERCA1a embedded in any of the single phospholipid species.

**Table 1:** Order of slope values in rate *vs* activity coefficient plots depicted in Fig. 4c.

Sample	Lipids		Order of slope values
Microsome			WT > R324E > R324A
Nanodiscs	Neutral	POPC	R324A ∼ WT > R324E
		POPE	R324A > WT ∼ R324E
	Acidic	POPS	WT > R324A > R324E
		POPG	WT > R324A > R324E
	Mixture		WT > R324E > R324A

## Discussion

The properties of the lipid bilayer surrounding the Ca^2+^-ATPase are crucial for pump function. The length of the fatty acid acyl chains strongly affects enzyme activity (a chain length of approximately C18–C16 is optimal) and, thus, the thickness of the lipid bilayer is critical^28,29^. In this study, 1-palmitoyl-2-oleoyl-glycerophospholipids were chosen for nanodisc reconstitution because a method for constructing nanodiscs harboring SERCA1a with these lipids had been already established^20^. The length of the acyl chains of these lipids (C16:0–C18:1) is optimum for SERCA1a; additionally, the specific effects of lipid headgroups could be compared by using phospholipids with the same acyl chains. In this paper, we used four types of phospholipids (Supplemental Fig. S6). The phospholipid content of the SR membrane includes mostly PC (68%) and smaller amounts of PE (16%) and phosphatidylserine (PS) (11%). The latter are asymmetrically located in the outer and inner leaflets of the SR membrane^22^: most PE (80%) is distributed in the outer (cytoplasmic) leaflet and PS (84%) in the inner (luminal) leaflet.

SERCA1a reconstituted in nanodiscs with this lipid composition could reproduce the order of the slope in the rate versus activity coefficient plots observed in microsomes (“triple” in Fig. 4c,d). In contrast, the affinities for luminal Ca^2+^ obtained from microsomes were only partially reproduced by the mixed-lipid samples (Fig. 2e). Nanodiscs cannot reproduce the asymmetric lipid distribution, so the proportion of PE facing Arg324 in this sample is lower than that in the SR membrane. This suggests that a higher PE content is necessary to reproduce the latter result. The lipid headgroups surrounding Ca^2+^-ATPase modulate the rate-limiting EP transition step via both electrostatic and non-electrostatic interactions^20^. A high content of PE in artificial liposomes causes a marked reduction in ATPase activity^30,31^. In contrast, the alteration of lipid composition induced by obesity results in inhibition of SERCA and progressive endoplasmic reticulum (ER) stress in hepatocytes^32^. Here, an increased PC/PE ratio in the ER may be the reason for SERCA inhibition and ER stress. These properties, together with the present results, suggest that a particular lipid composition of the membrane is critical for the physiological functions of SERCA.

Ca^2+^ binding involves the reactions E2 + 2Ca^2+^  ↔ E1Ca_2_ (scheme 1 in Fig. 3f, Supplementary Fig. S1, steps 1 and 2), which are accompanied by large changes in the arrangement of cytoplasmic domains and protein tilting. According to the available crystal structures, during this step, the electrostatic Arg324-lipid interaction breaks and the Arg324-OHTyr122 hydrogen bond forms. A simple scheme (scheme 2 in Fig. 3f) without considering H^+^ release and Mg^2+^ binding^24,25^ is sufficient to discuss our results described below (also see Supplemental discussion).

The affinity of all mutants (R324A, R324E, and Y122F) for cytoplasmic Ca^2+^ is identical to that of the wild-type (Fig. 2c and Supplementary Table S2). However, the Ca^2+^ release rates from E1Ca_2_ of R324A and Y122F are slower than those of the wild-type and R324E (Fig. 3b). Therefore, the binding of Ca^2+^ to E1 of R324A and Y122F must be slower than that of the wild-type and R324E. To explain this, assuming a pre-E1Ca_2_ state (*E1Ca_2_ in scheme 2–4 in Fig. 3f) seems mandatory. An intermediate form is supported by the finding that the time course of dissociation of bound Ca^2+^ is the same as that of the loss of EP formation activity (Fig. 3a,b). The lack of an Arg324-Tyr122 interaction would increase the transition energy of the *E1Ca_2_ ↔ E1Ca_2_ step and slow down Ca^2+^ release from E1Ca_2_ (scheme 3 in Fig. 3f).

On the other hand, the rate of EP formation from the Ca^2+^-free species upon Ca^2+^ addition is accelerated in the R324A and R324E mutants but not in Y122F (Fig. 3c–e). The Arg324-lipid interaction observed in the E2 structure is disrupted by the Ala substitution and becomes a repulsive interaction in the Glu substitution, resulting in destabilization of the E2 conformation. Therefore, it seems reasonable to consider that the transition rate from E2 to E1 is accelerated in these mutants (scheme 4 in Fig. 3f). Unchanged affinities for cytoplasmic Ca^2+^ indicate that the equilibrium between E2 and E1 was unaltered. The Arg324-lipid interaction stabilizes the E2 conformation and compensates for the strain emanating from driving the P-domain close to the membrane plane.

**Figure 5:**
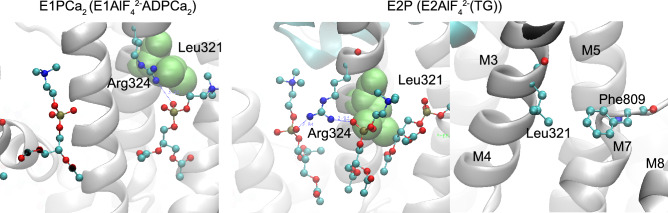
Location of Leu321 in crystal structures of E1PCa_2_ and E2P analogs. Left and middle panels, Arg324 and its connecting phospholipids are shown in the ball and stick model and Leu321 in van der Waals spheres in E1PCa (PDB ID: 5XA8) or an E2P analog (PDB ID: 5XA9). Right panel, Leu321 and Phe809 are shown in ball and stick model in an E2P analog (PDB ID: 5XA9). The transmembrane helices M3–5, 7, and 8 are indicated in the right panel.

The ATPase activities of wild-type and mutants were suppressed by low mM Ca^2+^ (Fig. 2b,d and Supplementary Table S1). This property reflects back-inhibition by luminal Ca^2+^ binding to vacant low affinity sites on the luminal side. This Ca^2+^ binding decreases the proportion of E2P and increases E1PCa_2_, thereby slowing the overall reaction. Apparent affinities followed the series R324E > R324A > WT ~ Y122F, indicating that the interaction between Arg324 and phospholipid is important in decreasing the affinity for luminal Ca^2+^. The opposite effect has been observed in a mutant of SERCA2b (L321F, where Leu321 is substituted by Phe) identified in a patient with Darier disease^33^, and found not to affect maximal ATPase activity but to drastically lower the Ca^2+^ affinity on the luminal side^34,35^. Leu321 lies just beneath Arg324 in the structure of the Ca^2+^-ATPase and is buried in lipid headgroups in the E1PCa_2_ and E2P structures (Fig. 5). It is close to Phe809, and its substitution by a phenylalanine may anchor the top of the M4 helix, including Arg324, to the near-membrane region via a π–π stacking interaction with Phe809. Thus, mutations in Arg324 (which eliminate anchoring) and Leu321 (which possibly increase anchoring) are detrimental in opposite ways; however, together they reinforce the notion that anchoring in this region has a direct effect on luminal Ca^2+^ affinity and hence pump function.

The particular affinity ranges for luminal Ca^2+^ observed in microsomes was only observed under a POPE environment when SERCA1a was embedded in nanodiscs. In SERCA1a embedded in POPC or POPS nanodiscs, the affinity of the wild-type for luminal Ca^2+^ was lower than that in microsomes, and was not affected by Arg324 mutations. These results suggest that there are additional lipid-protein interactions, other than those formed by Arg324, in a pure POPC or POPS environment that are eliminated by introducing POPE. In nanodiscs with a mixture of the three lipids, the affinity of the wild-type to luminal Ca^2+^ was similar to that in microsomes and not affected by the mutations, despite the identical percentage of POPE in the lipid mixture and in the SR membrane. This discrepancy may arise from the asymmetric distribution of PE in the outer and inner leaflets of the native membrane. In microsomes, PE may be enriched around SERCA1a compared with the average lipid components of microsomes. As mentioned above, obesity induces an alteration in lipid composition, which inhibits SERCA activity and causes ER stress in hepatocytes^32^. The key change is an increased PC/PE ratio in the ER. In line with the present results, the increasing PC/PE ratio (or decreasing PE contents) would decrease the affinity of SERCA for luminal Ca^2+^ and then perturb the Ca^2+^ homeostasis of the cell, causing dysfunction as seen in the L321F Darier disease patient.

In 0.1 M KCl, the order of EP transition rates among wild-type and mutants in microsomes (WT > R324A > R324E) was expected (Fig. 4a); the interaction between Arg324 and phospholipids must be eliminated by the substitutions, and Glu may repel neighboring phospho-headgroups and exacerbate the situation. However, analysis of the transition using a plot of the rate versus the square of the mean activity coefficient (Fig. 4b), where forces are separated into electrostatic and steric components, provides insights into the underlying mechanism, and the outcome can be counter-intuitive. In the more straightforward case of Ca^2+^-ATPase in microsomes, R324A has the least favorable electrostatic interactions (lowest slope), but smallest steric perturbation (highest intercept value); this combination results in the EP transition rate of R324A being slower than the wild-type but faster than R324E in 0.1 M KCl.

When Ca^2+^-ATPase is reconstituted in nanodiscs, the rate *vs* activity coefficient plots are quite different and show variable electrostatic and steric effects in various lipid environments (Fig. 4c,d and Supplemental Fig. S4). In the single-phospholipid environment, the order of the slope values, linked to the electrostatic contribution in the EP transition step, depends on the net charge of the headgroup (Supplemental Fig. S6). For acidic phospholipids (POPS and POPG), where the net charge is negative (the serine and glycerol moieties are effectively neutral), the order is WT > R324A > R324E. Here, in the wild-type protein, a phospho-Arg324 interaction may be relatively strong, so that an arginine-to-alanine change would have a large effect, and an arginine-to-glutamate change accompanied by repulsion even larger, explaining the observed order. On the other hand, the order of the slope values in neutral phospholipids (POPC and POPE) was R324A > WT > R324E (although the changes are small for R324A in POPC and R324E in POPE). In this case, in the wild-type protein, the phospho-Arg324 interaction may be weakened by the positive charge of the choline or ethanolamine moiety, making the effect of an alanine substitution less pronounced, but again heightened for a glutamate change with repulsion.

In microsomes, the slope value of R324A was smaller than that of R324E (Fig. 4b–d), in opposition to the values obtained for nanodiscs with any single type phospholipid. However, SERCA1a embedded in nanodiscs with a mixture of the three phospholipids (POPC, POPE, and POPS) showed the same order as in microsomes (WT > R324E > R324A). It appears that a complex process including formation and disruption of the Arg324-lipid interaction, to which different phospholipids contribute, is involved in the EP transition step of Ca^2+^-ATPase. In fact, during the EP transition step, the addition of a new ion-pair between Arg324 and the phosphate moiety of a phospholipid is observed in crystal structures^13^. It seems that the EP transition process includes exchange of lipid partners for Arg324, and formation of more than two interactions with different kinds of headgroups, or possibly both. Therefore, Ca^2+^ release from E2PCa_2_ may be optimal with a PE interaction, but the next step could favor other kind of phospholipid partner. Drastic rearrangements, such as Arg-PC → PE-Arg-PC → PE-Arg, may be occurring in the EP transition step.

## Conclusion

Arg324 of SERCA1a plays a role in the pump function by modulating Ca^2+^-site transitions. The EP transition step, in which Ca^2+^ is released to the lumen, is affected in a complex manner by Arg324 interactions with a variety of phospholipid residues. Our results cannot be explained by Arg324 interaction with a single species of phospholipid; the electrostatic contribution to the kinetics must arise from transient interactions with several phospholipid species. This strengthens the notion that phospholipids can play a direct role in the function of membrane proteins, and changes in lipid composition may affect their properties.

## Methods

### Materials

The pMSP1D1 plasmid (#20061) was purchased from Addgene (Watertown, MA, USA). The non-ionic detergent octaethylene glycol monododecyl ether (C_12_E_8_) was purchased from Tokyo Chemical Industry Co. LTD (Tokyo, Japan). 1-palmitoyl-2-oleoyl-sn-glycero-3-phospho-L-serine (sodium salt) (POPS) was purchased from Avanti Polar Lipids and other phospholipids (POPC, POPE, and POPG) from NOF Corporation (Tokyo, Japan). Lipids were dissolved in buffer containing 10 mM Tris/HCl (pH 7.5) and 100 mM (54 mg/ml) C_12_E_8_ and stored at − 80 °C. The membrane scaffold protein MSP1D1 was prepared as described previously^17,20^.

### Preparation of microsomes expressing wild-type and mutant SERCA1a from COS-1 cells

The cDNAs of full-length wild-type or mutant SERCA1a were inserted in the pMT2 expression vector^36^ as previously described.^15^ The vectors were transfected into COS-1 cells using Lipofectamine and PLUS reagent (Thermo Fisher Scientific, Waltham, MA, USA) according to the manufacturer's instructions. Microsomes were prepared from the cells as previously described^37,38^.

### Reconstitution of SERCA1a from microsomes in nanodiscs

Nanodiscs containing SERCA1a from COS-1 microsomes were prepared as previously described^20^. Microsomes (0.5 mg/ml) were incubated with 20 μM MSP1D1 in the presence of 3 mM phospholipid (POPC, POPE, POPS, or POPG) or a mixture of phospholipids (2.1 mM POPC, 0.55 mM POPE, and 0.35 mM POPS), 10 mM CaCl_2_, 20 mM Tris/HCl (pH 7.5), and 10 mg/ml C_12_E_8_ on ice for 30 min. To remove C_12_E_8_, the mixture was incubated with 0.4 mg/ml BioBeads SM2 (Bio-Rad) at 4 °C for 4 h with gentle agitation. The formed nanodiscs were centrifuged using a Vivaspin concentrator (100 kDa MWCO, GE Healthcare) to change the solution to 0.3 M sucrose, 0.1 M KCl, 0.1 mM CaCl_2_, 5 mM MOPS/Tris (pH 7.0) and to concentrate the sample. The samples were stored at − 80 °C after flash-freezing in liquid nitrogen.

### Ca^2+^-ATPase activity

The rate of ATP hydrolysis was determined at 25 °C in a mixture containing 10 μg/ml protein, 10 μM [*γ*-^32^P]ATP, 0.1 M KCl, 7 mM MgCl_2_, 1 mM EGTA, and 3 μM A23187 with various concentrations of CaCl_2_ (3 μM − 11 mM) to achieve the desired Ca^2+^ concentrations. The reaction was terminated with 0.1 M HCl, and the amount of ^32^P_i_ released from [*γ*-^32^P]ATP was quantified using digital autoradiography. The turnover rate was calculated based on the content of phosphorylation sites, which was determined for each sample according to the method described by Barrabin et al.^39^ with slight modifications. The samples (20 μg/ml protein) were phosphorylated with 10 μM [γ-^32^P]ATP in a buffer containing 30 mM Tris/HCl (pH 7.5), 80 mM KCl, 5 mM MgCl_2_, and 10 mM CaCl_2_ at 25 °C for 10 s, and the amount of EP thus formed was determined.

### Determination of EP

SERCA1a phosphorylation was performed as described in the figure legends, and the reaction was terminated with 7% TCA. The amount of EP was determined using digital autoradiography after separation by 5% SDS-PAGE at pH 6.0 according to Weber and Osborn^40^ as previously described^41^.

### Ca^2+^ release time courses from E1Ca_2_

Ca^2+^ release time courses from E1Ca_2_ were estimated from EP formation activity or bound Ca^2+^ at 4 °C. To measure bound Ca^2+^, microsomes (20 μg/ml) were incubated with 10 μM ^45^CaCl_2_ in the presence of 50 mM MOPS/Tris (pH 7.0), 0.1 M KCl, 7 mM MgCl_2_ and spotted on a membrane filter. Ca^2+^ release was initiated by washing the membrane with EGTA buffer containing 50 mM MOPS/Tris (pH 7.0), 0.1 M KCl, 7 mM MgCl_2_, and 1 mM EGTA. The membrane was then washed with buffer containing 50 mM HEPES/NaOH (pH 8.0), 0.1 M KCl, 7 mM MgCl_2_, 10 mM CaCl_2_, and 0.1 mM ATP at the indicated time points after EGTA washing to prevent further Ca^2+^ release from Ca^2+^-ATPase. After the final wash, the membranes were dried and the amount of ^45^Ca remaining on the membranes was measured. Simultaneously, the same procedure was performed in the presence of 1 μM thapsigargin (TG), a highly specific and subnanomolar affinity inhibitor of SERCA that blocks the enzyme in the Ca^2+^-unbound E2 state^42^. The amount of Ca^2+^ remaining on the membrane was subtracted from the amount of Ca^2+^ measured in the absence of TG and the amount of Ca^2+^ thus obtained was taken as Ca^2+^-ATPase specific Ca^2+^ binding. For estimation of Ca^2+^ release from EP formation activity, microsomes (20 μg/ml) were incubated with 10 μM CaCl_2_ in the presence of 50 mM MOPS/Tris (pH 7.0), 0.1 M KCl, and 7 mM MgCl_2_. Ca^2+^ release was initiated by addition of 2 mM EGTA. To phosphorylate the remaining E1Ca_2_, [γ-^32^P]ATP was added at the indicated time points after EGTA addition. Then the reaction was terminated with 7% TCA at 2 s after ATP addition, and the amount of formed EP was measured.

### Miscellaneous

Protein concentration was determined using the method described by Lowry et al*.*^43^ or the absorbance at 280 nm. Data were analyzed by nonlinear regression using the Origin software (Microcal Software, Inc.). Three-dimensional models of SERCA1a were produced using VMD^44^.

## Supplementary Information

Supplementary Information.

## Data Availability

All data generated and/or analyzed during the current study are available in this published article (and its Supplementary Information files).
