# Supplementary material for: Electrostatic interactions between single arginine and phospholipids modulate physiological properties of sarcoplasmic reticulum Ca2+-ATPase
Source: Sci Rep. 2022 Jul 16;12:12200. doi: 10.1038/s41598-022-16091-9 (PMC9288429; doi:10.1038/s41598-022-16091-9)
Supplement: Supplementary file 1 — Supplementary Information. [file 41598_2022_16091_MOESM1_ESM.pdf]

Supplementary Information for

Electrostatic interactions between single arginine and phospholipids modulate physiological properties of sarcoplasmic reticulum Ca^{2+}-ATPase

*Kazuo Yamasaki, Takashi Daiho, Satoshi Yasuda, Stefania Danko, Jun-ichi Kawabe and Hiroshi Suzuki

Department of Biochemistry, Asahikawa Medical University
078-8510 Midorigaoka-higashi 2-1-1-1, Asahikawa, Japan

*To whom correspondence should be addressed.
Kazuo Yamasaki
Department of Biochemistry, Asahikawa Medical University, Midorigaoka-higashi, Asahikawa, 078-8510, Japan,
Tel.: +81-166-68-2353; Fax: +81-166-68-2359
Email: kyamasak@asahikawa-med.ac.jp

This PDF file includes:

Figures S1 to S6
Tables S1 to S5
Supplemental discussion
SI References

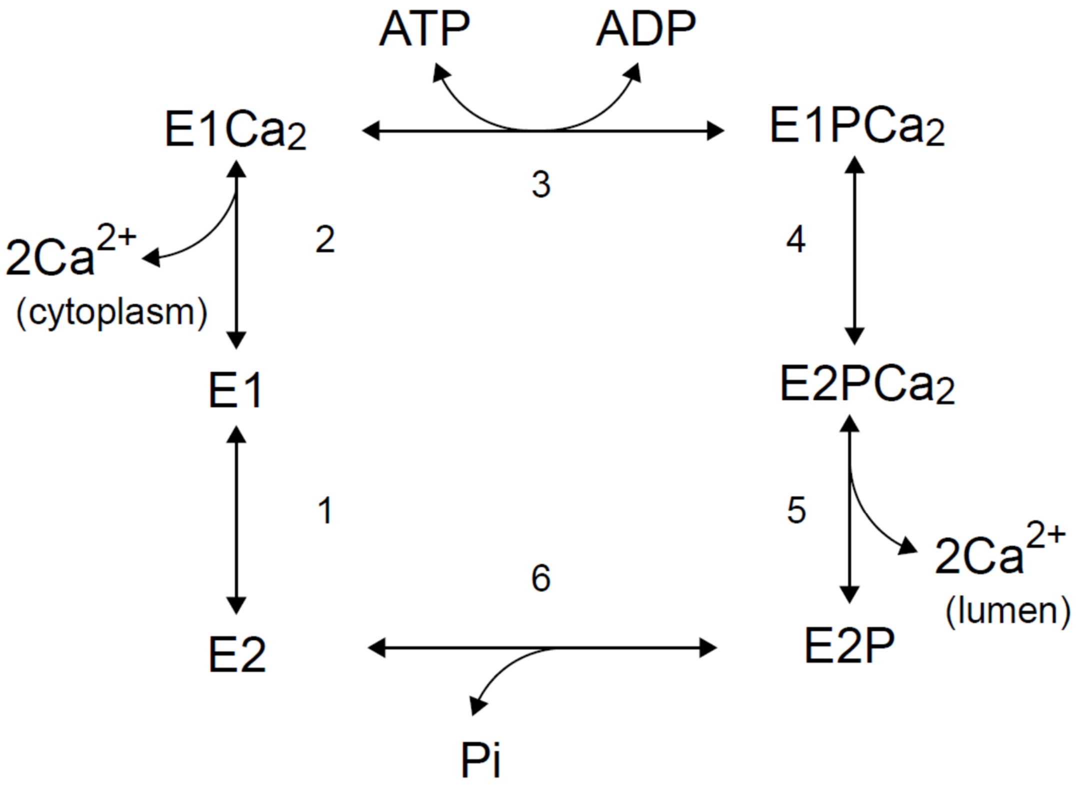

Figure S1. Reaction scheme of sarcoplasmic reticulum Ca^{2+}-ATPase.

SERCA1a transports Ca^{2+} ions from the cytoplasm to the lumen through coupling with ATP hydrolysis against a 1000-fold concentration gradient[1–3]. In the first step of the reaction cycle, the enzyme is activated by binding two Ca^{2+} ions from the cytoplasmic side with high affinity (< mM) (steps 1 and 2). Then, the enzyme is auto-phosphorylated by ATP and forms a phosphoenzyme intermediate (E1P, step 3). During formation of E1P, the cytoplasmic domains come together and the top part of the M4 helix connecting to the P domain bends towards the A domain, occluding the two bound Ca^{2+} ions (E1PCa2). The E1P phosphoenzyme intermediate spontaneously de-phosphorylates upon reaction with ADP to regenerate ATP in the reverse reaction, and is therefore called the "ADP-sensitive EP." Following an isometric phosphoenzyme transition step, EP looses ADP-sensitivity (forming an ADP-insensitive EP, E2P) as the TGES-loop on a rotated A-domain intrudes into the phosphorylation site (Asp351) located in a well-inclined P-domain. The Ca^{2+} binding sites now open towards the luminal side and exhibit low affinity (sub mM order) (step 4). The release pathway closes after dissociation of Ca^{2+} towards the lumen and a Ca^{2+}-unbound EP is formed (E2P, step 5). E2P hydrolyzes spontaneously to end the reaction cycle (step 6).

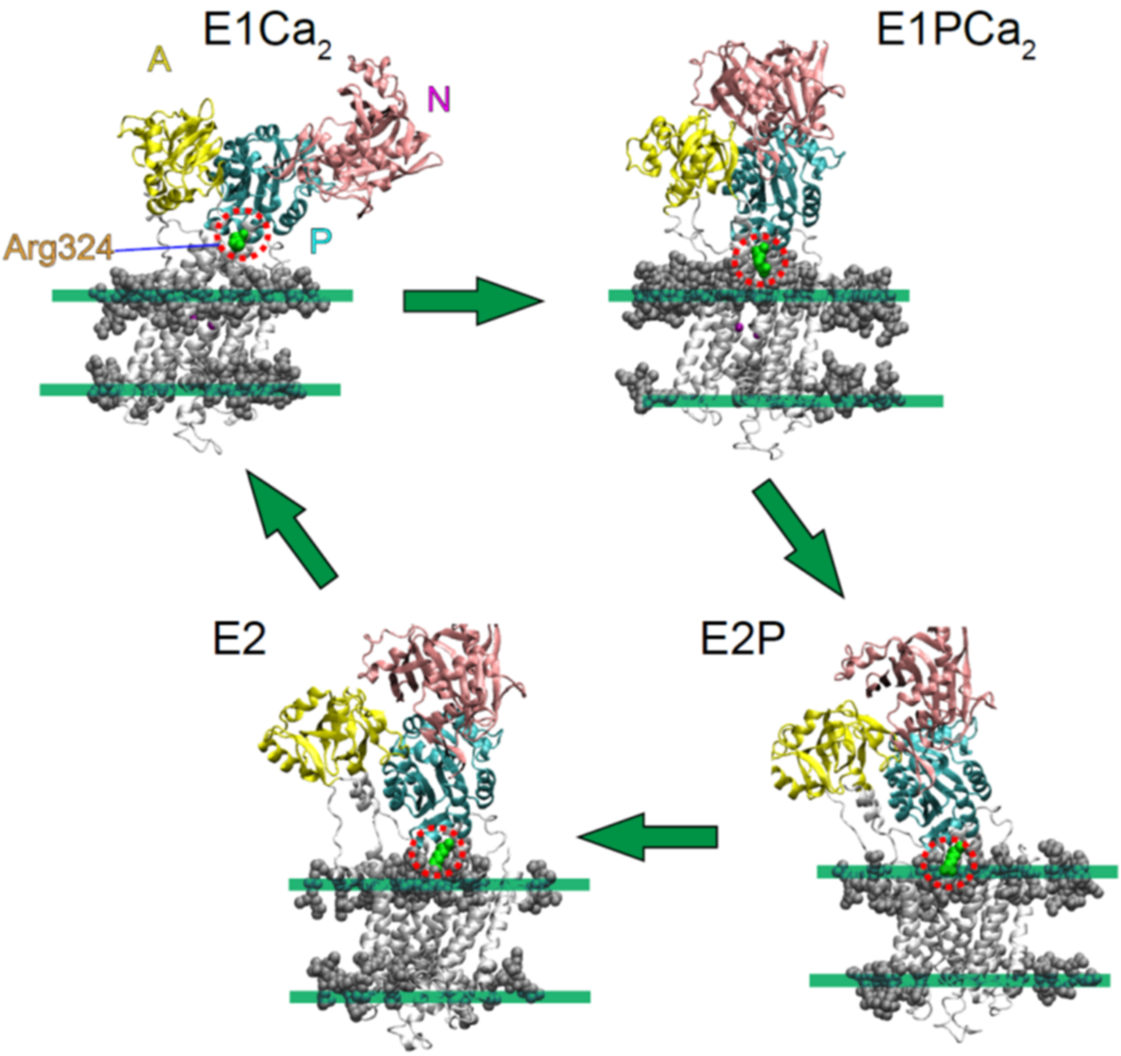

Figure S2. Crystal structures of SERCA1a and associated phospholipids.

The coordinates for the structures of E1Ca2, E1PCa2, E2P, and E2 with phosphatidylcholine were obtained from the Protein Data Bank (PDB IDs: 5XA7, 5XA8, 5XA9, and 5XAB, respectively). The cytoplasmic domains A, P, and N are colored in yellow, cyan, and pink, respectively, and transmembrane helices are shown in gray with the two bound Ca^{2+} ions in purple. The approximate position of the membrane region is shown by horizontal lines. The Arg324 residue drawn as green van der Waals spheres is highlighted with red dotted circles. The lipid headgroups observed in the structures are drawn as gray van der Waals spheres.

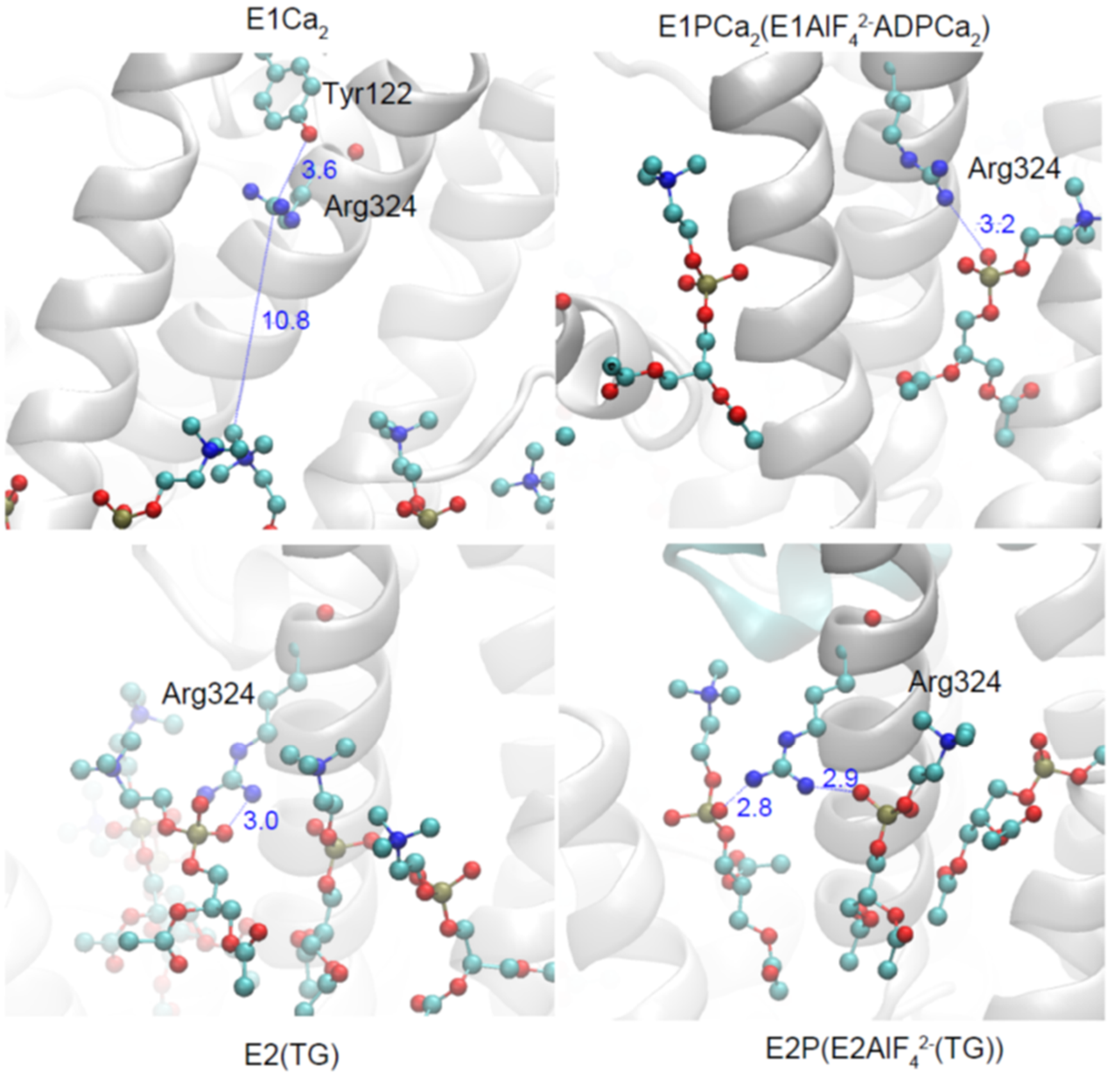

Figure S3. Interactions between Arg324 and phospholipids observed in crystal structures.

Arg324 and phospholipids observed in crystal structures (shown in Supplementary Fig. S2) are represented in the ball and stick model. The nearest atoms to Arg324 and lipids are linked by blue dotted lines. The distances are 10.6 Å (in $E1Ca_2$), 3.2 Å (in E1PCa analog), 2.9 or 2.8 Å (in E2P analog), and 3.0 Å (in E2 analog). Tyr122 is also shown in the $E1Ca_2$ structure. The distance between Tyr122 and Arg324 is 3.6 Å in $E1Ca_2$, but much shorter in another $E1Ca_2$ structure (PDB ID: 1SU4), around 2.9 Å.

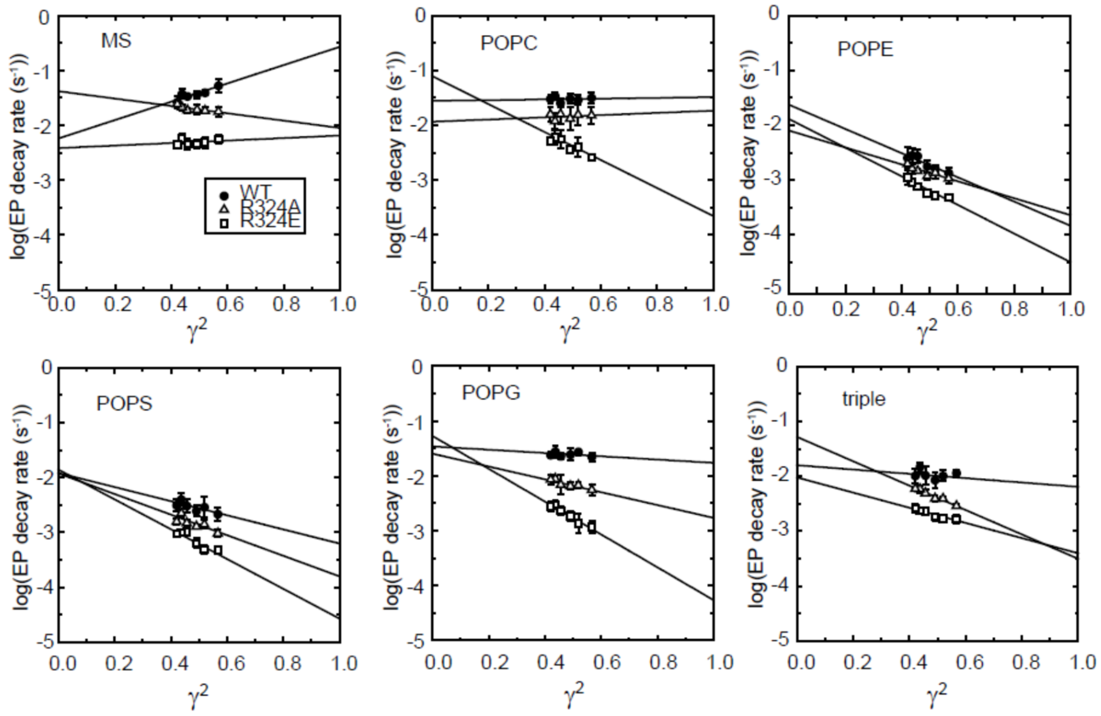

Figure S4. Rate versus activity coefficient plot of Ca^{2+}-ATPase embedded in nanodiscs. EP transition rates were determined with microsomes or nanodiscs harboring wild-type or mutant SERCA1a with various phospholipids (POPC, POPE, POPS, POPG or triple) and various KCl concentrations (as described in Fig. 4). Their logarithms are plotted against $\gamma_{\pm}^2$. The values presented are the mean ± standard deviation (n = 3–6). Solid lines show the least square fit in a linear regression; the fitting parameters, slope, and intercept at $\gamma_{\pm}^2 = 0$ are listed in Supplementary Table S4; the slope and intercept values are shown in Fig. 4c and Supplementary Fig. S5, respectively. The parameters are also listed in Supplementary Table S4. MS, microsomes; POPC, palmitoyl-oleoyl-phosphatidylcholine; POPE, palmitoyl-oleoyl-phosphatidylethanolamine; POPS, palmitoyl-oleoyl-phosphatidylserine; POPG, palmitoyl-oleoyl-phosphatidylglycerol; "triple," mixture of POPC, POPE, and POPS.

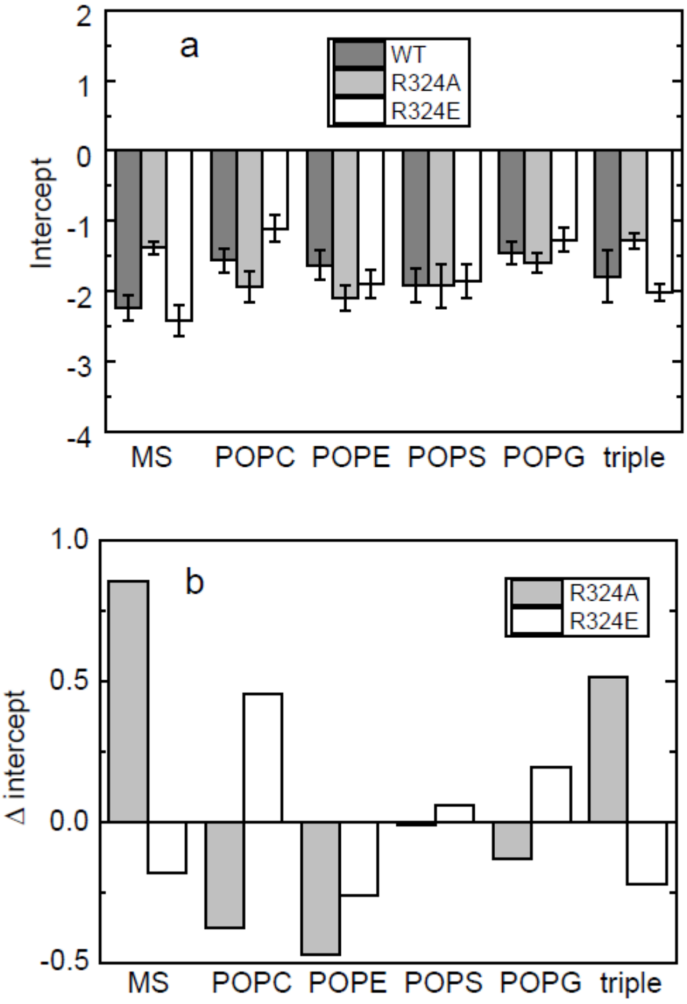

Figure S5. Intercepts from rate versus activity coefficient plot of Ca^{2+}-ATPase embedded in nanodiscs harboring various phospholipids.

(a) Intercepts obtained from Supplementary Fig. S4. The error bars indicate the standard error of the mean. The parameters are also listed in Supplementary Table S4. *(b)* The difference between the intercept of wild-type and mutants. WT, wild-type; MS, microsomes; POPC, palmitoyl-oleoyl-phosphatidylcholine; POPE, palmitoyl-oleoyl-phosphatidylethanolamine; POPS, palmitoyl-oleoyl-phosphatidylserine; POPG, palmitoyl-oleoyl-phosphatidylglycerol; “triple,” mixture of POPC, POPE, and POPS.

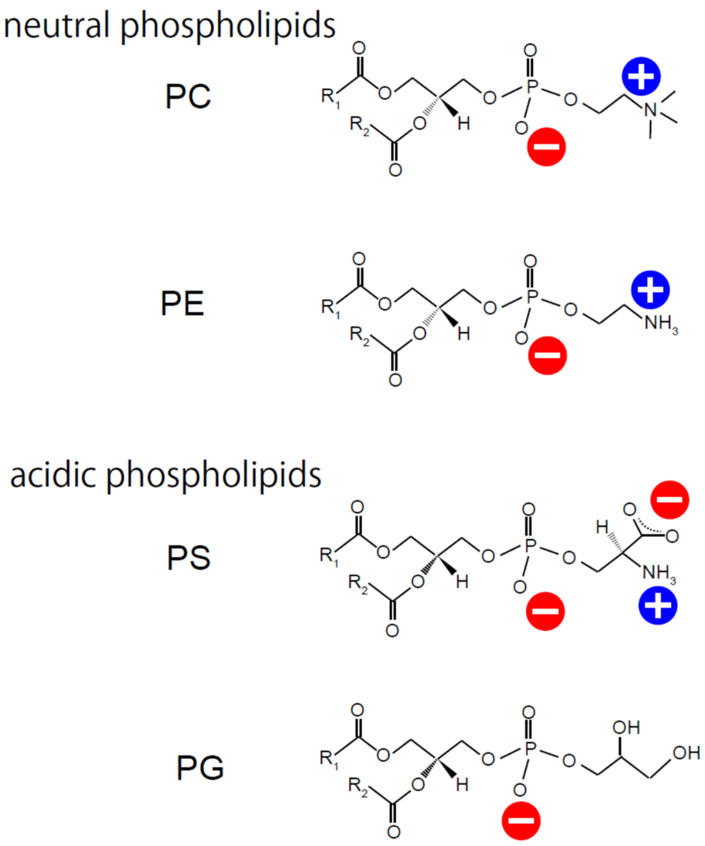

Figure S6. Structures of the phospholipids' headgroups used in this work.

In this paper, four types of phospholipids were used: phosphatidylcholine (PC), phosphatidylethanolamine (PE), phosphatidylserine (PS), and phosphatidylglycerol (PG). The headgroups of PC and PE possess a negative (phosphate) and a positive (choline or amino) groups, and thus net charges are neutral. In contrast, the headgroups of PS possess two negative (phosphate and carboxyl) and a positive (amino) groups, and thus the net charge is positive. PS possibly binds Ca^{2+} and mediates fusion of liposomes in the presence of over millimolar concentrations of Ca^{2+}. It is difficult to exclude the effects of PS-Ca^{2+} binding under such conditions (Fig. 2). However, the Hill coefficients (often accounted as the number of binding sites) of inhibition by high Ca^{2+} concentration in POPS was similar to those in the other lipid environments (Supplemental Table S5). Thus, we believe it is unlikely that PS-Ca^{2+} binding affects this step. PG has only one negative charge (phosphate group). Although the proportion of PG in the sarcoplasmic reticulum membrane is small, we used this lipid because it is possible to observe the effects of Arg324-phosphate interaction without perturbation by other charges in the headgroup.

Table S1. Fitting parameters for Fig. 2b

Ca^{2+} dependences of ATPase activities of microsomes (v) were fitted to the complex Hill equation as follows.

$$\boldsymbol{v = V_{max}\left(\frac{1}{1+\left(\frac{K_1}{[Ca^{2+}]}\right)^{n_1}}\right)\left(1-\frac{1}{1+\left(\frac{K_2}{[Ca^{2+}]}\right)^{n_2}}\right)}$$

Values indicate the fitted parameter ± standard error of the mean.

Microsome	K_1	n_1	K_2	n_2
	μM		*mM*	
WT	0.36 ± 0.07	1.2 ± 0.1	0.64 ± 0.04	1.8 ± 0.1
R324A	0.28 ± 0.04	1.2 ± 0.1	0.50 ± 0.04	1.6 ± 0.2
R324E	0.16 ± 0.02	1.3 ± 0.2	0.37 ± 0.03	1.6 ± 0.2
Y122F	0.28 ± 0.02	1.3 ± 0.1	0.60 ± 0.02	1.7 ± 0.1

Table S2. Fitting parameters for Fig. 2c

Affinity of microsomes for Ca^{2+} determined from EP formation activity. Values indicate the fitted parameter ± standard error of the mean.

Microsome	K	n
	μM	
WT	0.80 ± 0.03	2.7 ± 0.2
R324A	0.73 ± 0.03	2.0 ± 0.2
R324E	0.72 ± 0.04	1.8 ± 0.2
Y122F	0.88 ± 0.06	1.7 ± 0.2

Table S3. EP formation rate from Ca^{2+}-unbound species

The EP formation time courses in Fig. 3c and d were fitted to a single exponential increase. Values indicate the fitted parameter ± standard error of the mean. In parentheses, the values obtained with the wild-type are normalized to 1.

Microsome	EP formation rate	
	+Mg	-Mg
	s^{-1}	s^{-1}
WT	2.9± 0.2	3.1 ± 0.2
R324A	4.9 ± 0.3 (1.7)	5.7 ± 0.4 (1.8)
R324E	6.5 ± 0.4 (2.2)	7.4± 0.6 (2.4)
Y122F	2.2 ± 0.1 (0.8)	2.9 ± 0.2 (0.9)

Table S4. Slope and intercept in rate *vs* activity coefficient plots

The slope and intercept were obtained from the fitting of rate *vs* activity coefficient plots shown in Supplementary Fig. S4. Values indicate the fitted parameter ± standard error of the mean.

		Lipid	Slope	Intercept
microsome	WT		1.7 ± 0.4	-2.2 ± 0.2
	R324A		-0.7 ± 0.2	-1.4 ± 0.1
	R324E		-0.2 ± 0.4	-2.4 ± 0.2
nanodiscs	WT	POPC	0.1 ± 0.4	-1.6 ± 0.2
	R324A	POPC	0.2 ± 0.5	-1.9 ± 0.2
	R324E	POPC	-2.6± 0.4	-1.1 ± 0.2
	WT	POPE	-2.2 ± 0.4	-1.6 ± 0.2
	R324A	POPE	-1.5 ± 0.4	-2.1 ± 0.2
	R324E	POPE	-2.6 ± 0.4	-1.9 ± 0.2
	WT	POPS	-1.3 ± 0.5	-1.9 ± 0.2
	R324A	POPS	-1.9 ± 0.3	-1.9 ± 0.3
	R324E	POPS	-2.7 ± 0.5	-1.9 ± 0.2
	WT	POPG	-0.3 ± 0.8	-1.5 ± 0.2
	R324A	POPG	-1.2 ± 0.3	-1.6 ± 0.1
	R324E	POPG	-3.0 ± 0.3	-1.3 ± 0.2
	WT	triple	-0.4 ± 0.8	-1.8 ± 0.4
	R324A	triple	-2.2 ± 0.2	-1.3 ± 0.1
	R324E	triple	-1.4 ± 0.2	-2.0 ± 0.1

WT, wild-type; POPC, palmitoyl-oleoyl-phosphatidylcholine; POPE, palmitoyl-oleoyl-phosphatidylethanolamine; POPS, palmitoyl-oleoyl-phosphatidylserine; POPG, palmitoyl-oleoyl-phosphatidylglycerol; “triple,” mixture of POPC, POPE, and POPS.

Table S5. Values of Kd showed in Fig. 2e and corresponding Hill coefficients

Lipid			WT	R324A	R324E
MS	Kd	*mM*	0.82 ± 0.06	0.55 ± 0.10	0.34 ± 0.03
	n		1.8 ± 0.7	2.0 ± 0.5	1.6 ± 0.4
POPC	Kd	*mM*	1.03± 0.03	1.11 ± 0.18	1.01 ± 0.19
	n		1.6 ± 0.1	1.9 ± 0.5	1.8 ± 0.2
POPE	Kd	*mM*	0.64 ± 0.10	0.47± 0.12	0.37± 0.08
	n		2.3 ± 0.7	1.5 ± 0.1	1.1 ± 0.3
POPS	Kd	*mM*	0.94 ± 0.24	0.93 ± 0.23	1.06 ± 0.42
	n		1.6 ± 0.6	1.2 ± 0.5	1.3 ± 0.2
triple	Kd	*mM*	0.74 ± 0.03	0.68 ± 0.15	0.74 ± 0.04
	n		1.6 ± 0.4	1.3 ± 0.2	1.2 ± 0.2

MS, microsomes; POPC, palmitoyl-oleoyl-phosphatidylcholine; POPE, palmitoyl-oleoyl-phosphatidylethanolamine; POPS, palmitoyl-oleoyl-phosphatidylserine; “triple,” mixture of POPC, POPE, and POPS.

Supplemental discussion

The equilibrium between E1 and E2 is reported to be sensitive to pH and temperature, with most of the enzyme population in the E1 state at pH 7 and low temperature[4]. However, we have previously found that the EP formation rate from a Ca^{2+}-free species is increased two-fold in both R324A and R324E without changing the affinity for Ca^{2+} even at pH 6.0[5]. This means that there must exist a state preceding E1. The present data show that the EP formation rates from the Ca^{2+}-free species upon addition of Ca^{2+} and Mg^{2+} are not altered by preincubation with Mg^{2+} (Fig. 3c-e). Therefore, wild-type and all mutants must be in a Mg-free state at the start of the assay irrespective of the presence of Mg^{2+}. Furthermore, the faster EP formation rates of Arg324 mutants suggest the existence of an Arg324-lipid interaction in the Ca^{2+}-free state of wild-type. Therefore, we can assume that the Ca^{2+}-ATPase is in the E2 state (or in an E2-like structure) in the absence of Ca^{2+} at pH 7.0.

The discrepancy may be explained by the presence or absence of sarcolipin, a small membrane protein modulating the activity of SERCA[6,7]. In an E1Mg structure[8], sarcolipin is present in one of two SERCA chains in the crystallographic unit cell, and is located in a groove between the M2 and M4 helices. Arg324 forms a hydrogen bond with Glu2 of sarcolipin in this Mg-bound state. The protein increases the affinity of SERCA for Ca^{2+}, likely by altering the E2–E1 equilibrium towards E1. However, our sample does not contain sarcolipin because the SERCA was prepared from COS-1 cells lacking sarcolipin. Therefore, under the conditions of our assay, Ca^{2+}-free SERCA is probably in an E2 state even at pH 7 and low temperature.

SI References

1. Toyoshima, C. Structural aspects of ion pumping by Ca^{2+}-ATPase of sarcoplasmic reticulum. *Arch. Biochem. Biophys.* **476**, 3–11 (2008).
2. Toyoshima, C. How Ca^{2+}-ATPase pumps ions across the sarcoplasmic reticulum membrane. *Biochim. Biophys. Acta BBA - Mol. Cell Res.* **1793**, 941–946 (2009).
3. Møller, J. V., Olesen, C., Winther, A.-M. L. & Nissen, P. The sarcoplasmic Ca^{2+}-ATPase: design of a perfect chemi-osmotic pump. *Q. Rev. Biophys.* **43**, 501–566 (2010).
4. Pick, U. & Karlish, S. J. Regulation of the conformation transition in the Ca-ATPase from sarcoplasmic reticulum by pH, temperature, and calcium ions. *J. Biol. Chem.* **257**, 6120–6126 (1982).
5. Yamasaki, K., Daiho, T., Danko, S. & Suzuki, H. Multiple and Distinct Effects of Mutations of Tyr122, Glu123, Arg324, and Arg334 Involved in Interactions between the Top Part of Second and Fourth Transmembrane Helices in Sarcoplasmic Reticulum Ca2+-ATPase. *J. Biol. Chem.* **279**, 2202–2210 (2004).
6. Odermatt, A. *et al.* Characterization of the gene encoding human sarcolipin (SLN), a proteolipid associated with SERCA1: absence of structural mutations in five patients with Brody disease. *Genomics* **45**, 541–553 (1997).
7. Odermatt, A. *et al.* Sarcolipin Regulates the Activity of SERCA1, the Fast-twitch Skeletal Muscle Sarcoplasmic Reticulum Ca^{2+}-ATPase. *J. Biol. Chem.* **273**, 12360–12369 (1998).
8. Toyoshima, C. *et al.* Crystal structures of the calcium pump and sarcolipin in the Mg^{2+}-bound E1 state. *Nature* **495**, 260–264 (2013).
